# Abdominal Migraines: A Rare Adulthood Manifestation of a Typical Childhood Disease

**DOI:** 10.7759/cureus.36451

**Published:** 2023-03-21

**Authors:** Mohamed Ibrahim, Isaac Elkins, Michael Herman

**Affiliations:** 1 College of Osteopathic Medicine, Lake Erie College of Osteopathic Medicine, Bradenton, USA; 2 Gastroenterology, Borland Groover, Jacksonville, USA

**Keywords:** gut-brain axis, unexplained abdominal pain, irritable bowel syndrome, cyclic vomiting syndrome, migraine disorder

## Abstract

Abdominal migraine (AM) is a common childhood disease that rarely presents in adulthood. While multiple diagnostic guidelines have been established, AM can generally be described as unprovoked episodes of acute central abdominal pain with migrainous features and periods of relief. AM is believed to be caused by a disturbance in the gut-brain axis. We are presenting a case of a 47-year-old Caucasian female with a six-month history of abdominal pain and vomiting. The episodes occurred one to two times per week, for 12-18 hours. These episodes were unprovoked and the patient felt normal in between episodes. Her past medical history is notable for hypertension and childhood migraines. Extensive imaging and laboratory workups were unremarkable. A trial of as-needed 50-milligram sumatriptan was started. The patient’s symptoms were aided and became less frequent over the next three months. Although uncommon, this patient’s case presents convincing evidence of AM. Cyclic vomiting syndrome (CVS), another disease of gut-brain access, was once thought to be a pediatric disease. However, further research showed relevant prevalence in the adult population. CVS has a similar mechanism and treatment plan to AM. It seems plausible that a closely related gut-brain axis disorder like AM could have more prevalence in the adult population. To better identify AM in adults, it is important that physicians inquire about a history of childhood migraines when faced with vague abdominal symptoms. Increased identification of AM will help guide treatment and improve patient outcomes.

## Introduction

Abdominal migraine (AM) is a functional disorder without a defined pathologic mechanism or a biochemical irregularity that typically presents in childhood and adolescence [1]. Children often present with intermittent bouts of severe central abdominal pain with migrainous attributes such as sensory hypersensitivities to light and sound, vomiting, nausea, acute headaches, and general pallor [1]. The majority of children often overcome the condition by their teen years and do not have any lasting developmental or neurological problems; however, a small percentage of patients carry these symptoms into adulthood [1]. While the prevalence in children has been reported as high as 9.2% in a 2016 questionnaire study of the mothers of 949 children, adult AM diagnosis is extremely rare and just a few case reports have been published thus far [1]. One of the few studies done on this illness by Roberts and deShazo was a cohort of 13 patients where 10 met some criteria for AM; however, 90% of them had a strong family history of migraines [2]. Their findings, along with other literature, suggest including AM in the differential diagnosis of patients who present with periodic abdominal pain and migrainous features [2]. In this case report, we introduce a patient who presented with nausea, vomiting, and abdominal pain with a known childhood history of migraines.

## Case presentation

A 47-year-old Caucasian female with a past medical history of hypertension and childhood migraines presented to the outpatient gastroenterology clinic with a six-month history of recurrent abdominal pain and associated nausea and vomiting. Episodes would occur randomly at least one to two times a week and last for 12-18 hours. She reported no related symptoms between episodes and no initiating events or activities that provoked the incidents. There was no association with eating or drinking. There were no new medications added or changes in lifestyle to prompt such attacks. Laboratory data, which included a complete blood count, basic metabolic panel, drug screening, thyroid-stimulating hormone level, and morning cortisol level, were all within normal limits. A computed tomography (CT) scan of the abdomen, ultrasound of the gallbladder, hepatobiliary iminodiacetic acid (HIDA) scan, and upper gastrointestinal endoscopy were also all normal and showed no abnormalities. Her past medical history was notable for hypertension and childhood migraines, which stopped at the age of 17. The patient was started on 50 milligrams of sumatriptan as needed at the onset of migraine symptoms for the possible diagnosis of AM. Her symptoms resolved with treatment and became less frequent over the next three months. While other metabolic syndromes and biochemical disturbances must be ruled out, there is a recognized criterion for the diagnosis of AM as defined by the International Classification of Headache Disorders (ICHD-3beta) [3], Rome IV Classification of Gastrointestinal Disorders [4], Symon and Russel Definition of Abdominal Migraine [5], and presented in 2018 by Angus-Leppan et al. in Table 1 [1]. With all her laboratory values being unremarkable, a past medical history of childhood migraines, relief with the use of sumatriptan, and using the criteria in Table 1, a clinical diagnosis of exclusion of AM was warranted.

**Table 1:** Recommended Pragmatic Definition of Abdominal Migraine Adapted from definitions of Symon and Russel [5], International Classification of Headache Disorders (ICHD-3beta) [3], and Rome IV [4]

Recommended Pragmatic Definition of Abdominal Migraine
Episodic central abdominal pain, usually lasting >1 hour
Episodes interfere with normal activity
Episodes occur with one or more of pallor, anorexia, nausea, vomiting, photophobia, or headache, or are associated with other episodic syndromes (particularly cyclical vomiting and migraine limb pain)
Person is well between episodes
Normal physical and developmental examination

## Discussion

While AM was traditionally considered a childhood disease that subsided by the teenage years, the manifestations of this illness in adults could give merit to its recurrence in later years. The cause of AM in children is highly hypothesized, but some suggest that a combination of changes to the gut-brain relationship, vascular dysregulation, changes in the central nervous system, as well as genetic factors may play a big role in the development of this disease in childhood and adulthood, just as in other forms of migraines [1]. With a focus on the gut-brain axis, the enteric nervous system (ENS) is one of three subcategories of the autonomic nervous system with the other two divisions being the sympathetic and parasympathetic as defined by the British physiologist, John Newport Langley [6]. The complexity of the highly innervated ENS has allowed it to exhibit integrative neuronal activity with the capacity to control gastrointestinal behavior and movement with very little to no input from the brain or spinal cord [6]. It contains more neurons than the entirety of the peripheral ganglia and a likely disconnection between the gut and central nervous system could be the source of association between migrainous features and abdominal symptoms [2,6]. In 2016, Devanarayana et al. elaborated on the role of the ENS in AM when they studied the gastric motility of patients with AM compared to a control group and showed that gastric and antral motility was significantly decreased in those children with AM [2]. They also noticed a strong correlation between migraine severity and lower gastric motility, giving rise to the idea that the gut-brain axis is more prevalent than once anticipated and that central innervation of the gut may be the root source of AM [2]. Furthermore, variations in gut permeability were also proven in AM patients. In a small study of 20 children, 11 abdominal migraine patients and nine control patients, Bentley et al. showed that there was an increase in mucosal permeability of the small intestine in AM patients when compared to the control patients [2]. After following three AM patients, they were also able to show a significant improvement in symptoms when the gut permeability decreased and ultimately resolved [2]. Although children with AM have a great prognosis and the majority show complete remission of symptoms with time, these initial changes during the development of the gut-brain axis could help us understand how they are related to migraines and how they may manifest and present in adulthood.

To further support the prevalence of AM in adults, we can turn to examples of related pathology. Cyclic vomiting syndrome (CVS) is another gut-brain axis pathology characterized by episodes of emesis with periods of remission. CVS was thought to be primarily a pediatric disease associated with childhood migraines [7]. It was later realized that CVS occurs in adults [8]. With sumatriptan being a successful treatment for CVS and AM exacerbations [9,10], we ponder if CVS and AM could be mechanistically related. Like AM, one mechanism underlying CVS is gastric dysmotility [11]. Interestingly, CVS is primarily associated with increased motility, whereas AM is primarily associated with decreased motility [2,7]. This possible relationship between CVS and AM is important because it demonstrates that once seemingly exclusive pediatric diseases of the gut-brain axis are either lingering on in adulthood or manifesting for the first time. With both patient populations exhibiting histories of childhood migraines, this common link should be further explored. Table 2 below explains some of the similarities and differences between AM and CVS [1,2,7-11].

**Table 2:** Comparisons of Abdominal Migraines and Cyclic Vomiting Syndrome Treatments may vary on a case-by-case basis. Cases of both abdominal migraines and cyclic vomiting syndrome have been successfully aborted with sumatriptan [9,10], supporting a possible link in pathology.

Brain-Gut Axis Pathology	Abdominal Migraines	Cyclic Vomiting Syndrome
Primary Onset	Childhood	Childhood
Pattern of Disease	Episodic, Unprovoked	Episodic, Unprovoked
Associated Medical Diagnosis	Childhood Migraines	Childhood Migraines
Associated GI Findings	Decreased GI Motility	Increased GI Motility
Successful Treatment*	Sumatriptan	Sumatriptan
Prevalence in Adults	Unknown	Established

Once a diagnosis is made, treatments for AM should be explored. There are multiple options available for AM patients. The patient in our case was successfully treated with sumatriptan. However, there have been cases of AM failing treatment with sumatriptan [10]. There has been success in treating AM with prophylactic agents used for conventional migraine headaches. These other options include beta-blockers, calcium channel blockers, topiramate, valproate, amitriptyline, and cyproheptadine [10]. Many cases have been successfully managed with a prophylactic agent and an abortive agent [10]. With such a wide array of medications available, physicians should be able to successfully tailor treatment to minimize side effects for their patients.

Due to the nonspecific symptoms and the overlap between AM and many other gut-brain axis diseases, such as CVS, we emphasize a thorough workup and detailed patient history in order to connect this childhood illness with adult patient clinical manifestations. Our case presentation is not without limitations. While the patient had intermittent migraines that were relieved by sumatriptan, no neurological workup or brain scans were explored in order to rule out central nervous system causes of migraines. The lack and scarcity of case reports and research papers explaining how abdominal migraines could potentially develop in adults is another limitation that hinders our ability to expand on what seems to be a more common but overlooked disease due to the presumption that it is only a childhood condition. We urge clinicians and gastroenterologists to put AM on their differential list when a patient presents with irritable bowel syndrome (IBS)-like symptoms such as altered stool consistency, altered stool frequency, bloating, and abdominal distention, along with associated migrainous features such as headaches, vomiting, and nausea.

## Conclusions

Abdominal migraines (AM) are unprovoked episodes of central abdominal pain associated with migraine symptoms such as sensory hypersensitivities to light and sound, vomiting, nausea, acute headaches, and general pallor. While common in children, AM is considered a rare diagnosis in adults. Our case demonstrates the possibility that AM could be more prevalent in adults than currently recognized. Our patient, a 47-year-old Caucasian female, fits the current diagnostic criteria for AM, and her symptoms were reduced with sumatriptan. Importantly, her past medical history was notable for childhood migraines. The example of cyclic vomiting syndrome (CVS) demonstrates that former gut-brain axis diseases of childhood can be more prevalent in the adult population when further researched. With a similar mechanism of pathology, we believe that AM could be more prevalent in the adult population. To identify AM in adults, it is imperative that physicians know the association between childhood migraines and AM. When faced with vague abdominal symptoms, this could be a key clue that could help lead to a diagnosis and successful treatment. To further our understanding of the childhood gut-brain axis occurring in adults, the pathophysiology linking CVS and AM with past childhood migraines should be explored.
